# Specific Host Signatures for the Detection of Tuberculosis Infection in Children in a Low TB Incidence Country

**DOI:** 10.3389/fimmu.2021.575519

**Published:** 2021-03-15

**Authors:** Alexandra Dreesman, Véronique Corbière, Myriam Libin, Judith Racapé, Philippe Collart, Mahavir Singh, Camille Locht, Françoise Mascart, Violette Dirix

**Affiliations:** ^1^Laboratory of Vaccinology and Mucosal Immunity, Université Libre de Bruxelles, Brussels, Belgium; ^2^Pediatric Department, Centre Hospitalo-Universitaire Saint-Pierre, Brussels, Belgium; ^3^Biomedical Research Center, Erasme Hospital, Brussels, Belgium; ^4^Centre de recherche Epidémiologie, Biostatistiques, Recherche Clinique, School of Public Health, Université Libre de Bruxelles, Brussels, Belgium; ^5^Lionex Diagnostics and Therapeutics, Braunschweig, Germany; ^6^Univ. Lille, CNRS, Inserm, CHU Lille, Institut Pasteur de Lille, U1019 – UMR9017 – CIIL – Center for Infection and Immunity of Lille, Lille, France

**Keywords:** tuberculosis, purified-protein derivative, culture-filtrate-protein-10, heparin-binding haemagglutinin, tumor-necrosis-factor-α, interferon-γ-induced protein 10, Interferon-γ

## Abstract

Diagnosis of tuberculosis (TB) in children remains challenging due to unspecific clinical presentation and low bacillary load. In low TB incidence countries, most cases are diagnosed by a contact screening strategy after exposure to an index TB case. Due to the severity of TB in young children, the priority is to determine whether a child is infected or not, whereas differential diagnosis between active TB (aTB) and latent TB constitutes a second step. In Belgium, a low TB incidence country, we prospectively included 47 children with a defined *M. tuberculosis* infection status (12 children with aTB, 18 with latent TB, and 17 uninfected) (exploratory cohort), and determined the optimal combinations of cytokines secreted by their peripheral blood mononuclear cells in response to a 5-days *in vitro* stimulation with four different mycobacterial antigens, in an attempt to classify the children according to their infectious status. Correct identification of all infected children was obtained by several combinations of two purified protein derivative (PPD)-induced cytokines (IFN-γ and either GM-CSF, MIP-1α, sCD40L or TNF-α), or by combining PPD-induced IFN-γ with culture-filtrate protein-10 (CFP-10)-induced TNF-α. Alternatively, combining CFP-10-induced TNF-α and IP-10 with heparin-binding haemagglutinin (HBHA)-induced-IFN-γ was more effective in testing recently BCG-vaccinated children or those suspected to be infected with non-tuberculous mycobacteria, providing a correct classification of 97% of the *M. tuberculosis-*infected children. This combination also correctly classified 98% of the children from a validation cohort comprising 40 *M. tuberculosis* infected children and 20 non-infected children. Further differentiation between aTB and children with latent TB was more difficult. Combining ESAT-6-induced MIP1-α and IP-10, CFP-10-induced MIG, and HBHA-induced MIG provided a correct classification of 77% of the children from the exploratory cohort but only of 57.5% of those from the validation cohort. We conclude that combining the measurement of 2–4 cytokines induced by three different mycobacterial antigens allows an excellent identification of *M. tuberculosis-*infected children, whereas differentiating children with aTB from those with latent TB remains far from perfect.

## Introduction

Despite the implementation of effective chemotherapy more than 60 years ago, active tuberculosis (aTB) is still the leading cause of mortality from an infectious disease worldwide, with more than 10 million new cases and more than 1.4 million deaths each year (1). Among the new aTB cases, 1 million were diagnosed in children, and more than half of them were younger than 5 years of age in 2017 (1). Two hundred thirty-three thousand of these children with aTB died from it, 80% of which were younger than 5 years old (2), and these figures are most probably underestimated. Diagnosis of aTB in children is challenging, due to non-specific clinical presentation, low bacillary load, and difficulties of specimen collection. Diagnosis is therefore often made presumptively on the basis of a combination of symptoms, clinical signs, radiological findings and the results of the tuberculin skin test (TST). Sensitivity and specificity of these approaches are imperfect. Therefore, improved screening tests are urgently needed to diagnose *Mycobacterium tuberculosis* infection in children.

Like for adults, pediatric *M. tuberculosis* infection can result in latent TB infection (LTBI) or in aTB. LTBI is defined as a *M. tuberculosis* infection without clinical or radiological signs of disease, whereas in aTB, the child presents clinical signs and/or radiological abnormalities. In low TB-incidence countries, this distinction is often arbitrary in children who usually acquire infection through exposure to an adult TB index case and are diagnosed by a contact screening strategy. They are mostly pauci-symptomatic even when presenting with clear aTB. The priority, at least in young children, is therefore to determine whether a child is infected, as in the absence of treatment young infected children, despite the paucibacillary nature of the disease, often rapidly develop a severe form of aTB. This is one of the reasons why the development of triage and screening tests for children figures among the WHO key priorities (3). Differentiation between children with LTBI and those with aTB is a second challenge for pediatricians in order to appropriately decide treatment options.

Several immunological tests were developed to screen for infection in children. The first and oldest test is the TST. More recently, interferon-γ-release assays (IGRA) were developed to avoid possible false positive results of the TST in BCG-vaccinated children or in patients infected with non-tuberculous mycobacteria (NTM). However, none of these tests allows differentiation between aTB and LTBI. In addition, the performance of current commercial IGRA to detect LTBI appears to be less reliable than previously assumed (4), and the sensitivity of the IGRAs to detect aTB is not different from that of the TST when all age groups are considered (5). A negative IGRA test cannot rule out aTB. However, WHO recommends to develop “rule out tests” (6). Several other immunological approaches were therefore evaluated and reported in the literature. One of them combines the measurement of different cytokines released in the blood in response to mycobacterial antigens to increase the sensitivity of the IGRA that only measure IFN-γ concentrations (7–11).

The aim of this study was to determine an optimal combination of cytokines secreted in response to four different mycobacterial antigens to identify of *M. tuberculosis*-infected children in a low TB incidence country.

## Materials and Methods

### Ethics Statement

The study protocol (number P2011/113 and A2012/051) was approved by Comité d'Ethique hospitalo-facultaire Erasme - ULB, Brussels, Belgium, and informed written consent was obtained from all parents.

### Study Subjects

Sixty-one 0–15 years old children living in a low TB incidence country (Belgium) and recently exposed to an aTB index case (sputum smear positive adult) were included in a previous study analyzing the *M. tuberculosis*-specific T cell responses at the cellular level by flow cytometry (12). Fifteen of them presented with aTB, 19 were considered as being LTBI and 27 of them were non-infected. Because of the high complexity of flow cytometry, we decided to evaluate the diagnostic potential of measuring secreted cytokine concentrations by multiparameter-based immunoassays on remaining supernatants from cell cultures. We prioritized inclusion of samples from children with an undoubtful diagnosis of aTB or LTBI and limited the numbers of samples from non-infected children to enroll comparable numbers of children in each group. We therefore arbitrarily included the 17 first available samples from the 27 non-infected children. In addition, as samples from the initial study were no longer available for six children, four with aTB and two with LTBI, samples from two infected children were added in this study, one from a child with aTB and another from a child with LTBI. Samples from 47 children were thus included in this study and they constituted the samples for the exploratory cohort (cohort 1). Samples from a second, independent cohort of children were tested afterwards, representing a validation cohort. This cohort comprised 60 children included in a study evaluating the ability of antigen-induced blast cells as determined by flow cytometry, to identify and correctly classify *M. tuberculosis*-infected children (13). This was a whole blood assay, and an IGRA in response to mycobacterial antigens was performed in parallel on PBMC (14, 15). Cell culture supernatants from these IGRA were included in this study for 60 children selected by chronological order of inclusion in the first study, 20 with aTB, 20 considered as being LTBI and 20 being non-infected.

The criteria applied to define aTB were the presence of symptoms consistent with aTB (chronic cough, persistent fever, night sweats, otherwise unexplained weight loss), radiological findings suggestive of aTB, TB exposure history, positive TST, positive microbiological results (*n* = 5 and *n* = 13 positive culture or polymerase chain reaction for the first and second cohort, respectively), or response to anti-tuberculous therapy in the absence of positive microbiological results (*n* = 7 and *n* = 7 for the 1st and 2nd cohorts). LTBI children were defined by a positive TST in an exposed child, without clinical or radiological signs of active disease, and non-infected children were asymptomatic with a negative TST up to 8–12 weeks after last contact with the aTB index case. The positivity of the TST was defined as an induration of at least 5 mm, or of at least 15 mm in case of previous BCG vaccination (16) 48 h after the intradermal injection of tuberculin (2 IU PPD RT23, Statens Serum Institute, Copenhagen, Denmark). The main demographic and clinical data from the children included in the two cohorts are described in Table 1. The study was conducted and is reported in accordance with Quality Assessment of Diagnostic Accuracy Studies (QADAS) criteria (17).

**Table 1:** Demographic and clinical data from the exploratory and validation cohorts.

	**aTB**		**LTBI**		**Non-infected**	
	**Exploratory**	**Validation**	**Exploratory**	**Validation**	**Exploratory**	**Validation**
	**cohort**	**cohort**	**cohort**	**cohort**	**cohort**	**cohort**
*N*	12	20	18	20	17	20
Median age (range) (yrs)	2 (0–14)	2 (0–15)	5.5 (2–15)	9 (0–14)	2 (0–8)	0.4 (0–4)
Gender - Female (%)	42	30	55	45	40	35
**Ethnic origin [*****n*** **(%)]**						
Caucasian	0 (0)	4 (20)	4 (22)	6 (30)	2 (12)	3 (15)
North-African	4 (33)	9 (45)	5 (28)	10 (50)	7 (41)	9 (45)
Sub-Saharan African	4 (33)	5 (25)	2 (11)	3 (15)	0 (0)	4 (20)
Other/mix/unknown	4 (33)	2 (10)	7 (39)	1 (5)	8 (47)	2 (10)
BCG vaccinated [*n* (%)]	0*	0**	5 (28)	2 (10)	1 (6)	2 (10)
***M. tuberculosis*** **infection status**						
**- TST results**						
Positivity (%)	100	85°	100	100	0	0
Range of induration (mm)						
BCG +	/	/	15–25	13–15	0	0
BCG −	15–28	7–30	10–28	10–25	0	0
Abnormal chest X Ray (%)	92	100	0	0	0	0
Microbiology [Smear+ and/or culture+ and /or PCR+ (%)]	42	65	NA	NA	NA	NA
Response to TB treatment (%)	100	100	NA	NA	NA	NA
Pulmonary TB (%)	92	75°°	NA	NA	NA	NA

*aTB, active tuberculosis; LTBI, latently TB infected; TST, tuberculosis skin test; BCG, Bacille Calmette-Guerin, PCR, polymerase chain reaction; NA, non applicable*.

*1 unknown; **2 unknown; °% positive TST at treatment initiation; °°75% (ganglio) pulmonary TB, 25% extra-pulmonary and/or disseminated TB with associated chest X ray abnormalities.

### Cell Isolation and Stimulation

Heparinized blood was collected from all eligible children, and the samples were processed as described (12, 14, 15). Briefly, purified peripheral blood mononuclear cells (PBMC) were incubated at 37°C (5% CO_2_) with either 4 μg/ml PPD (Statens Serum Institute, Copenhagen, Denmark), 10 μg/ml ESAT-6 (Lionex, Diagnostics & Therapeutics GmbH, Braunschweig, Germany), 10 μg/ml CFP-10 (Lionex) or 10 μg/ml HBHA (purified from *Mycobacterium bovis* BCG as detailed elsewhere) (18). Cells incubated in antigen-free medium served as negative controls and cells incubated with staphylococcal enterotoxin B (SEB, Sigma-Aldrich, Bornem, Belgium; 0.5 μg/ml) served as positive controls. After 5 days *in vitro* incubation, supernatants were collected and cryopreserved at −20°C for batched analysis.

### Cytokine Analysis

The concentrations of 18 different host markers were measured in the supernatants using a multiparameter-based immunoassay: granulocyte macrophage colony-stimulation factor (GM-CSF), Interferon-gamma (IFN-γ), interleukin (IL)-2, IL-6, IL-10, IL-13, IL-15, IL-17A, IL-21, IL-23, IFN-γ-induced protein 10 (IP-10), Monocyte chemo-attractant protein-1 (MCP-1), Monokine induced by gamma interferon (MIG), macrophage inflammatory protein (MIP)-1α, MIP-1β, regulated upon activation normally T-cell expressed and secreted (RANTES), soluble CD40 ligand (sCD40L), and Tumor-necrosis-factor-alpha (TNF-α). The cytokine/chemokine concentrations were measured using Milliplex human cytokine/chemokine kits (Merck, Belgium) according to the manufacturer's instructions. Culture supernatants were diluted using dilution factors specific for each analyte in order to obtain concentrations within an interpretable range at least in the supernatants from PBMC *in vitro* stimulated with mycobacterial antigens. Results were analyzed with a Bio-Plex® MAGPIX^TM^ Multiplex reader, Bio-Plex Manager^TM^ MP Software and Bio-Plex Manager 6.1 Software (BIO-RAD laboratories, Nazareth Eke, Belgium). Standard curves were established to determine cytokine concentrations and only results obtained within the linear parts of the standard curves were considered for further analysis. Moreover, to ensure robustness of the results, we considered only those equal or higher than 10 pg/ml, even if the lower limit of detection was for most markers 4 pg/ml. This limit of 10 pg/ml was arbitrarily chosen with the aim of avoiding interpretation of statistical differences with improbable biological significance and results in a concentration range with possible high inter-run variability. The concentrations below this arbitrary limit were allocated a value of 10 pg/ml to allow statistical analysis with continuous variables, whereas results exceeding the assay's upper limit of detection were attributed the concentration corresponding to this limit. When detectable, the analyte concentrations obtained in the antigen-free conditions were subtracted from those obtained with antigen stimulation. These background cytokine secretions are reported in Supplementary Table 1 (exploratory cohort) and Supplementary Table 2 (validation cohort). The laboratory scientists performing the sample analysis were blinded to the clinical and other laboratory data from the children.

### Statistical Analyses

Host marker concentrations were represented by medians and percentiles (P25-P75), as the results probably did not follow a Gaussian distribution. Differences between groups of children were analyzed using the Mann-Whitney U test. The diagnostic abilities of individual host markers were assessed by receiver operator characteristics (ROC) curve analysis (GraphPad Prism version 7.03, GraphPad Software, La Jolla CA, www.graphpad.com). Relevance of the host marker selection for combined analysis was confirmed by random forest analysis using random Forest package version 4.6-14 (R-3.5.0, R Foundation for Statistical Computing, Vienna, Austria). The predictive abilities of combinations of host markers were investigated by logistic regression followed by a ROC curve via STATA version 14. Results were graphically represented with the linear predictor of the logistic regression. Statistically significant differences were determined using *p* < 0.05.

## Results

### Single Parameter for the Identification of *M. tuberculosis-*Infected Children

As the identification of *M. tuberculosis*-infected children (LTBI/aTB) is the first step of *M. tuberculosis* contact management in childhood, we evaluated the diagnostic potential to differentiate infected from non-infected children of the measurement of cytokine/chemokine concentrations released in cell culture supernatants of PBMC after *in vitro* stimulation with four different mycobacterial antigens (PPD, ESAT-6, CFP-10 and HBHA). The aim of this approach was to evaluate whether measurement of the concentration of one or several cytokines/chemokines may be used as a triage test to rule out infection.

**Figure 1:**
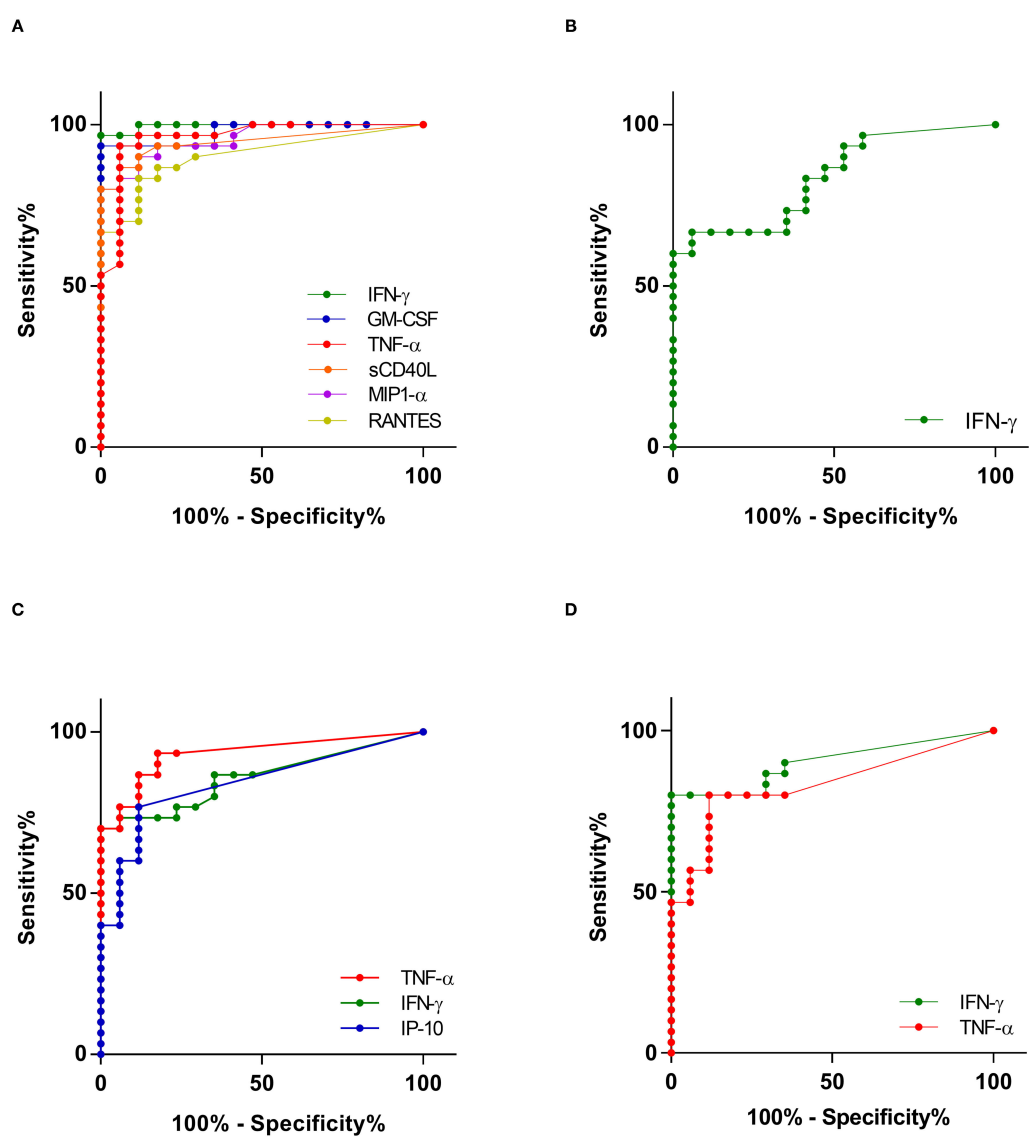
Receiver operating characteristic (ROC) curves showing the accuracies of top individual host markers in detecting *M. tuberculosis* infection. Only ROC curves for antigens that differentiated between infected and non-infected children with area under the curve (AUC) above 0.9 for PPD **(A)** and 0.8 for ESAT-6 **(B)**, CFP-10 **(C)**, and HBHA **(D)** are shown.

IL-2, IL-10, IL-15, IL-21, IL-23, and MCP-1 were discarded from the analysis, as the concentrations obtained for these cytokines/chemokines provided no significant differences between infected and non-infected children for the four mycobacterial antigens used to induce their secretion (Supplementary Table 3, *p* > 0.05). For the other 12 cytokines/chemokines, median concentrations induced by one or several mycobacterial antigens were significantly different between infected and non-infected children (Supplementary Table 3). However, substantial overlaps existed between the cytokine concentrations measured in samples from infected and those from non-infected children (Supplementary Table 3), so that we arbitrarily defined three criteria to further select the best host markers to identify *M. tuberculosis*-infected children: (1) the comparison of the cytokine concentrations between infected and non-infected children by the Mann-Whitney U test should provide a *p*-value of at least 0.0001 (Supplementary Table 3), (2) the area under the ROC curve established by comparing results from infected children to those from non-infected children should be higher than 0.9 for PPD, and higher than 0.8 for ESAT-6, CFP-10 and HBHA (Supplementary Table 3 and Figure 1), and (3) at least a 10-fold difference between infected and non-infected children should be noticed for the medians of cytokine concentrations (Supplementary Table 3).

Only six cytokines/chemokines fit these 3 criteria for PPD-induced secretion: IFN-γ, GM-CSF, TNF-α, sCD40L, MIP-1α and RANTES (Supplementary Table 3 and Figure 1A). The best discrimination between infected and non-infected children was obtained by comparing PPD-induced IFN-γ and GM-CSF concentrations that provided sensitivities of 97 and 93%, respectively, for the identification of infected children, with specificities of 100% for both cytokines. Only IFN-γ was selected for ESAT-6 *in vitro* stimulation of the PBMC, but it provided a poor sensitivity (67%) to identify infected children with a specificity of 94% (Figure 1B). Three host markers were selected for their induction by CFP-10, TNF-α, IFN-γ and IP-10, and the best results to identify infected children was obtained with TNF-α, which provided a sensitivity of 77% with a specificity of 94% (Figure 1C). Finally, both IFN-γ and TNF-α were considered relevant when induced by HBHA, both cytokines providing 100% specificity, and HBHA-induced IFN-γ allowed the detection of infection with a sensitivity of 80% (compared to 47% for TNF-α) (Figure 1D).

Random forest analysis performed on PPD-, ESAT-6-, CFP-10- or HBHA-induced host markers provided the same selection as that based on the three arbitrarily fixed criteria described above (Supplementary Figure 1).

### Combination of Multiple Host Markers for the Identification of *M. tuberculosis-*Infected Children

Logistic regression analysis was performed to evaluate the added value of combining several cytokine/chemokine concentrations secreted in response to a specific mycobacterial antigen for the identification of *M. tuberculosis*-infected children.

Different combinations comprising 3 or 4 PPD-induced host markers provided 100% correct classification of the children from the first cohort, but this performance was already obtained by combining only two parameters: PPD-IFN-γ/GM-CSF, PPD-IFN-γ/MIP-1α, PPD-IFN-γ/sCD40L, or PPD-IFN-γ/TNF-α. In response to CFP-10, combining IFN-γ and TNF-α concentrations slightly increased the sensitivity to detect infected children from cohort 1, from 77 to 80%. Finally, in response to HBHA, IFN-γ concentrations remained the best parameter with no added value of a combination with HBHA-induced TNF-α concentrations.

The accuracy of two of the best combinations, PPD-IFN-γ/MIP-1α and PPD-IFN-γ/TNF-α was evaluated on the validation cohort (Supplementary Table 4). The results were similar to those obtained in the exploratory cohort with 95% of the children from the validation cohort being well-classified by these combinations. Only three infected children were misclassified by both combinations.

**Figure 2:**
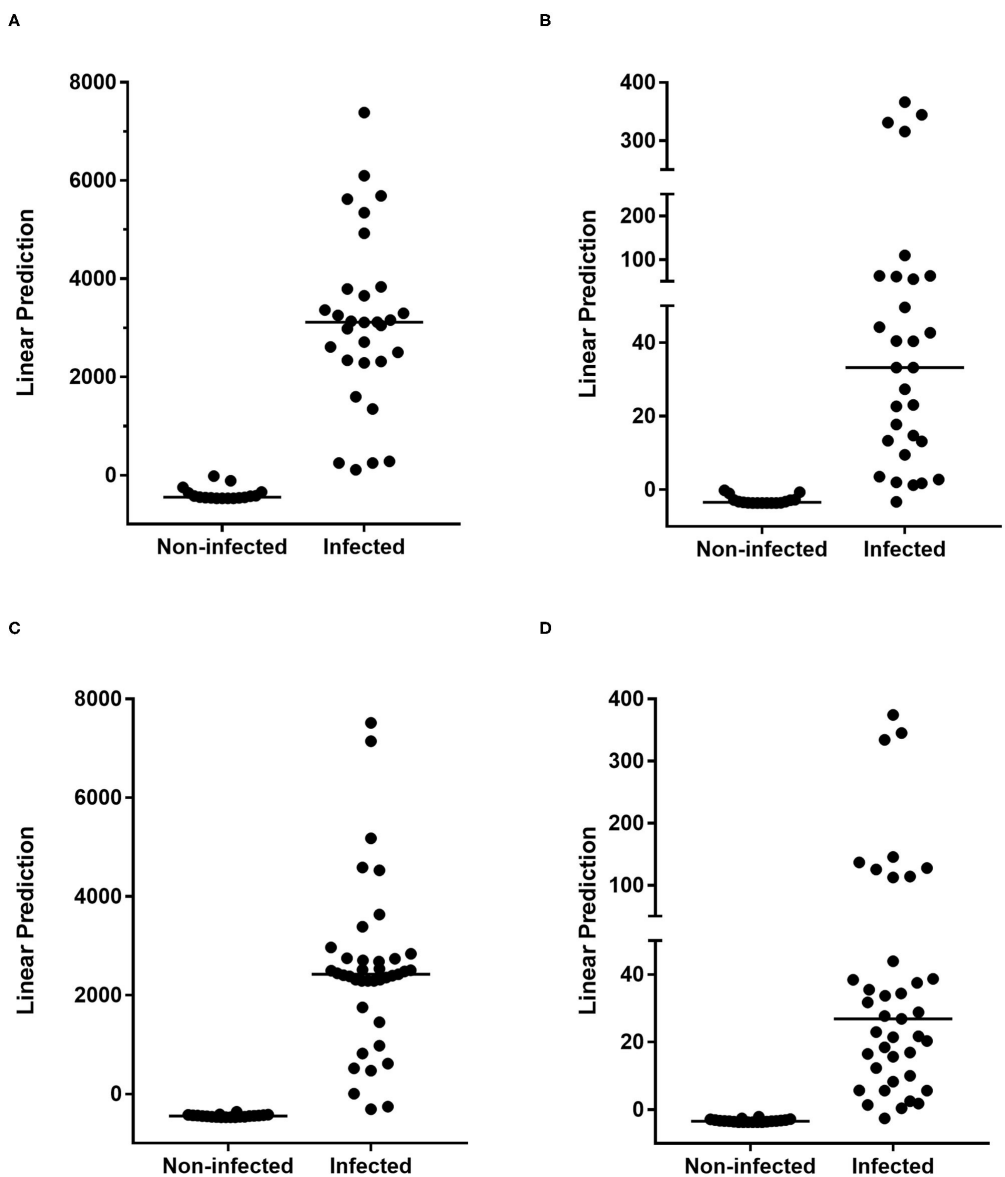
Combinations of *M. tuberculosis*-specific host markers allowing the distinction between *M. tuberculosis*-infected vs. non-infected children. PPD-IFN-γ and CFP-10-TNF-α **(A,C)** or CFP-10-TNF-α, CFP-10-IP-10, and HBHA-IFN-γ **(B,D)** combinations selected by logistic regression analysis are represented with their linear predictor for the exploratory **(A,B)** and the validation **(C,D)** cohorts. The horizontal lines represent the medians of the linear prediction values.

Logistic regression analysis was performed to further select the best host marker combinations induced by different mycobacterial antigens. The best combination was determined to be PPD-IFN-γ combined to CFP-10-TNF-α. Results represented for the exploratory cohort by their linear prediction value confirmed the optimal detection of *M. tuberculosis*-infected children by this combination that correctly classified all the children as infected or non-infected, with a very high median linear prediction in *M. tuberculosis-*infected children compared to non-infected children (Figure 2A). *M. tuberculosis*-infected children were thus identified by this combination with 100% sensitivity and 100% specificity. However, this combination might not be appropriate for recently BCG-vaccinated children or in case of suspicion of NTM infection. We therefore evaluated other host marker combinations, discarding PPD. In this case, the combination of three markers, CFP-10-TNF-α, CFP-10-IP-10, and HBHA-IFN-γ, resulted in a correct classification of 98% of the children. Only one infected child was misclassified providing 97% sensitivity with 100% specificity for the identification of *M. tuberculosis*-infected children (Figure 2B).

To confirm these results, a similar logistic regression analysis was performed with the same selected parameters on results obtained for the validation cohort. In this case, PPD-IFN-γ combined to CFP-10-TNF-α correctly classified 97% of the children, providing a sensitivity of 95% and a specificity of 100% for the identification of *M. tuberculosis*-infected children (Figure 2C and Supplementary Table 4). The combination without PPD-induced cytokines, CFP-10-TNF-α, CFP-10-IP-10, and HBHA-IFN-γ, provided a correct classification of 98% of the children from the validation cohort, the infected children from this cohort being identified with 98% sensitivity and 100% specificity (Figure 2D and Supplementary Table 4). Only one infected child was misclassified, like it was the case for the exploratory cohort, thus confirming the very high accuracy of this combination of markers to identify *M. tuberculosis*-infected children.

### Discrimination Between Children With LTBI and Those With aTB

Among the 18 cytokines measured in the supernatants after stimulation of PBMC with mycobacterial antigens, significant differences between children with LTBI and aTB were observed only when comparing the concentrations of PPD- and HBHA-induced IL-17A, CFP-10-induced IL-2, ESAT-6-induced IL-13, MIP-1α, MIP-1β, and RANTES (Supplementary Table 5). Significant *p-*values ranged from 0.02 to 0.05, and the best area under the ROC curve was 0.74, illustrating the substantial overlaps of the results between the two groups.

To select the most relevant markers for further logistic regression analysis, we calculated ratios between the medians of cytokine concentrations in the group of children with LTBI and those with aTB: a ratio >1 indicated higher values in children with LTBI, whereas a ratio <1 characterized children with aTB (Supplementary Figure 2). Whereas, most cytokines were more abundantly secreted by the PBMC from children with LTBI than from those with aTB, namely PPD-induced MCP-1, ESAT-6-induced MIP-1α, CFP-10-induced MCP-1 and HBHA-induced MIG, children with aTB were characterized by high concentrations of ESAT-6-induced IP-10 and of CFP-10-induced MIG (CXCL9) (Supplementary Figure 2). Logistic regression analyses were performed on the results obtained for these six selected markers, but no model reached a satisfying classification of the children as aTB vs. LTBI. The best results were obtained for the exploratory cohort by combining ESAT-6-MIP-1α and IP-10, to CFP-10-MIG and HBHA-MIG, and this provided a correct classification of 77% of the children. The accuracy of this combination of markers to differentiate children with aTB from those with LTBI was evaluated in the validation cohort, and only 58% of the children were correctly classified, confirming the poor discrimination between children with aTB and those with LTBI by the selected combination of markers.

## Discussion

The urgent development of triage or screening tests for children exposed to an aTB index case, or for children suspected to be infected with *M. tuberculosis* figures among the WHO key priorities in order to rule out infection or to use confirmatory tests in case of positive triage-tests (3). The minimal sensitivity of such tests for children has been set by the WHO at 90%, with a minimum specificity of 70% (3). By measuring the concentrations of different cytokines and chemokines secreted by PBMC in response to four different mycobacterial antigens and by comparing the results obtained for *M. tuberculosis*-infected children to those obtained for non-infected children, we identified here different host markers and combinations of host markers that fulfill these requirements. The measurements of PPD-induced IFN-γ concentrations provided 100% specificity and 97% sensitivity for the identification of *M. tuberculosis*-infected children, confirming previous reports (11, 16). A marginal increase in sensitivity (100%) was obtained by combining PPD-induced IFN-γ concentrations with one other host marker, such PPD-induced GM-CSF, MIP-1α, sCD40L or TNF-α, or CFP-10-induced TNF-α. However, as these performances were reached in a low TB incidence country with a low BCG vaccine coverage and a low incidence of NTM infection, they may not be applicable for countries with a systematic BCG vaccine strategy and/or high NTM infection incidence. Other, PPD-independent host marker combinations were therefore also evaluated. The combination of CFP-10-induced TNF-α and IP-10 concentrations with HBHA-induced IFN-γ concentrations provided 97% sensitivity with 100% specificity to identify *M. tuberculosis*-infected children in the exploratory and 98% sensitivity with 95% specificity in the validation cohort. This combination of cytokines missed only one infected child in each cohort. By comparison, the TST detected all the infected children from the exploratory cohort but was negative at treatment initiation for three children with aTB from the validation cohort, these three children being in contrast detected as infected by the combination of cytokines. This combination of three different cytokines secreted in response to two different mycobacterial antigens may therefore be proposed as a good triage test for children to be evaluated for a possible infection with *M. tuberculosis*. However, when possible, this approach should remain associated with a TST for a “no risk” approach.

Children with a positive triage test need to be further evaluated to discriminate those with LTBI from those with aTB to allow clinician to make appropriate and early therapeutic decisions. This cannot be achieved with the current diagnostic tests, neither the TST nor the commercial IGRA (19). Different approaches to this issue were reported and generally investigated on small cohorts of children. The measurement of different cytokines released after *in vitro* stimulation of whole blood with mycobacterial antigens, such as PPD or ESAT-6 and CFP-10, highlighted in a small cohort of children older than 10 years of age the potential interest of PPD-induced secretions of both TNF-α and IL-10, that classified all patients correctly (11). Another pilot study performed on six children with LTBI and eight children with aTB suggested a possible discrimination between the two groups by the ratio between the concentrations of TNF-α and IL-2 induced by ESAT-6 and CFP-10, with a low ratio in favor of LTBI (88% sensitivity and 83% specificity) (9). A high number of IL-2-secreting cells induced by the mycobacterial antigen Rv2780 (secreted L-alanine dehydrogenase), was reported to help identifying children with aTB among >5 years old children (8). Other approaches are based on the analysis by flow cytometry of cytokine-containing lymphocytes after *in vitro* stimulation with mycobacterial antigens. Promising results reported lower expression of CD27 by IFN-γ-containing CD4^+^ T lymphocytes in children with aTB compared to those with LTBI and uninfected controls (20). We previously demonstrated that the ratio between the proportions of ESAT-6-induced IFN-γ- and TNF-α-containing CD4^+^ T lymphocytes provided an excellent discrimination between LTBI and aTB among 3–15 years-old children, whereas for children younger than 3 years the differentiation between these two groups was better when the proportion of HBHA-induced IL-17 ^+^ CD4^+^ T lymphocytes were taken into account (12). Globally, none of these approaches was simple enough to be applied at an individual level by clinicians, and none of them provided a clear distinction between children with aTB and those with LTBI.

In the present study, we investigated the potential of 18 different host markers produced in response to four different mycobacterial antigens to correctly differentiate children with LTBI from those with aTB. The results obtained in the exploratory cohort of 30 infected children suggested that a combination of four host markers could provide some discrimination between LTBI and aTB. These results were not confirmed by testing a larger validation cohort of 40 infected children, confirming that differential diagnosis between LTBI and aTB in children based on immune biomarkers remains extremely difficult if at all possible. This challenge has never been met until now, at least on large cohorts, and this may be inherent to the pathogenesis of childhood TB and to the reference algorithm used to classify the children as LTBI or aTB. Most children classified as presenting aTB are culture negative for *M. tuberculosis*. Therefore, the classification remains partially subjective.

The results reported here on the chemokine secretions in *M. tuberculosis*-infected children indicated that all the HBHA-induced chemokines/cytokines were more abundantly secreted in children with LTBI than in those with aTB. These results are in line to those previously reported for the HBHA-induced IFN-γ secretion in adults, indicating that the HBHA-induced cellular immune responses are associated with latency (21, 22). Similar results were obtained for ESAT-6-induced chemokines with the exception of IP-10 that was more secreted in case of aTB than in case of LTBI. Interestingly, the profile of CFP-10-induced chemokines was rather different from that of ESAT-6-induced chemokines, even though the genes encoding these two proteins are both located in the RD-1 region of the *M. tuberculosis* genome. Different chemokines were preferentially induced by CFP-10 in children with aTB compared to those with LTBI, like previously reported for IFN-γ (14).

The strength of this study relies in the prospective evaluation of the diagnostic value of different immune parameters to identify *M. tuberculosis*-infected children among children exposed to a TB index case, in two independent cohorts of children. In contrast to most reported studies, we included here relatively large cohorts with a total of 32 children with aTB and 38 children with LTBI who were compared to 37 non infected children. All these children were living in a low-TB incidence country and only children responding to strict diagnostic criteria were taken into consideration for the final analyses. This was done in accordance with a strict diagnostic algorithm provided in Belgium as the recommended standard (16).

We conclude that easy-to-measure biomarkers fulfill the WHO requirements as a “triage test” to identify *M. tuberculosis*-infected children within a population of potentially infected children, and that they should ideally be performed in addition to the TST to fulfill a “no risk” approach. In contrast, the accurate differential diagnosis between children with aTB and those with LTBI remained far from perfect.

## Data Availability Statement

The raw data supporting the conclusions of this article will be made available by the authors, without undue reservation.

## Ethics Statement

The studies involving human participants were reviewed and approved by Comité d'Ethique Hospitalo-Facultaire - Erasme - ULB. Written informed consent to participate in this study was provided by the participants' legal guardian/next of kin.

## Author Contributions

AD acquired clinical data, interpreted the data, and approved the final manuscript as submitted. VC designed the study, analyzed and interpreted the data, critically reviewed the manuscript, and approved the final manuscript as submitted. ML performed the analysis and approved the final manuscript as submitted. JR performed the statistical analysis of the data and approved the final manuscript as submitted. PC performed the statistical analysis, critically reviewed the manuscript and approved the final manuscript as submitted. MS prepared antigens and approved the final manuscript as submitted. CL prepared antigens, critically reviewed and revised the manuscript and approved the final manuscript as submitted. FM designed the study, interpreted the data, drafted the final manuscript and approved its final version as submitted. VD designed the study, analyzed and interpreted the data, drafted the initial manuscript and approved the final manuscript as submitted. All authors contributed to the article and approved the submitted version.

## Conflict of Interest

MS was employed by the company Lionex Diagnostics and Therapeutics. The remaining authors declare that the research was conducted in the absence of any commercial or financial relationships that could be construed as a potential conflict of interest.
