# Supplementary material for: Specific Host Signatures for the Detection of Tuberculosis Infection in Children in a Low TB Incidence Country
Source: Front Immunol. 2021 Mar 15;12:575519. doi: 10.3389/fimmu.2021.575519 (PMC8005539; doi:10.3389/fimmu.2021.575519)
Supplement: Supplementary file 1 [file Table_1.pdf]

Supplementary Table 1. Background of cytokines in unstimulated condition (exploratory cohort).

	Median [P25-P75]		Median [P25-P75]		Median [P25-P75]
**GM-CSF**	45 [30-80]	**IL-15**	10 [10-10]	**MIG**	12109 [4150-33495]
**IFN-γ**	26 [13-54]	**IL-17A**	10 [10-15]	**MIP-1α**	10 [10-300]
**IL-2**	25 [15-60]	**IL-21**	10 [10-10]	**MIP1-β**	80 [55-125]
**IL-6**	10 [10-10]	**IL-23**	10 [10-10]	**RANTES**	495 [155-700]
**IL-10**	20 [10-30]	**IP-10**	20476 [6600-34000]	**sCD40L**	90 [56-180]
**IL-13**	25 [10-95]	**MCP-1**	30000 [16000-30000]	**TNF-α**	150 [75-275]

Results of the measured concentrations are reported in pg/ml as medians and 25th – 75th percentiles.
