# Supplementary material for: Specific Host Signatures for the Detection of Tuberculosis Infection in Children in a Low TB Incidence Country
Source: Front Immunol. 2021 Mar 15;12:575519. doi: 10.3389/fimmu.2021.575519 (PMC8005539; doi:10.3389/fimmu.2021.575519)
Supplement: Supplementary file 2 [file Table_2.pdf]

Supplementary Table 2. Background of cytokines in unstimulated condition (validation cohort).

	Median [P25-P75]
**IFN-γ**	10<br>[10-39]
**IP-10**	4311<br>[2133-9154]
**MIG**	6577<br>[2021-15893]
**MIP-1α**	27<br>[10-47]
**TNF-α**	73<br>[32-131]

Results of the measured concentrations are reported in pg/ml as medians and 25th – 75th percentiles.
