# Supplementary material for: Specific Host Signatures for the Detection of Tuberculosis Infection in Children in a Low TB Incidence Country
Source: Front Immunol. 2021 Mar 15;12:575519. doi: 10.3389/fimmu.2021.575519 (PMC8005539; doi:10.3389/fimmu.2021.575519)
Supplement: Supplementary file 3 [file Table_3.pdf]

Supplementary Table 3. PPD-, ESAT-6-, CFP-10-, and HBHA-induced cytokines (exploratory cohort).

Host-marker	PPD				ESAT-6				CFP-10				HBHA			
	Median [P25-P75]		p*	Area under ROC curve	Median [P25-P75]		p*	Area under ROC curve	Median [P25-P75]		p*	Area under ROC curve	Median [P25-P75]		p*	Area under ROC curve
	Non-infected	Infected			Non-infected	Infected			Non-infected	Infected			Non-infected	Infected		
**GM-CSF**	375 [40-1085]	4813 [2730-6470]	<0.0001	0.9765	350 [10-1638]	1777 [190-2995]	0.0408	0.6804	85 [10-333]	1138 [111-2865]	0.0004	0.8	25 [10-145]	271 [94-423]	0.0001	0.8216
**IFN-γ**	127 [10-395]	19973 [19050-19986]	<0.0001	0.9961	90 [10-713]	13300 [180-19972]	<0.0001	0.8343	10 [10-150]	4093 [113-19946]	<0.0001	0.8578	10 [10-35]	874 [241-2608]	<0.0001	0.901
**IL-2**	10 [10-10]	10 [10-10]	0.8239	0.5078	10 [10-10]	10 [10-10]	0.7455	0.5118	10 [10-10]	10 [10-12]	0.4539	0.549	10 [10-10]	10 [10-21]	0.1974	0.5892
**IL-6**	2920 [1355-5925]	13020 [7310-34760]	0.0001	0.8507	6105 [140-19800]	3760 [325-33355]	0.7876	0.5259	890 [140-1432]	1255 [330-4788]	0.1611	0.6247	140 [140-140]	140 [140-345]	0.003	0.7167
**IL-10**	10 [10-10]	10 [10-10]	>0.9999	0.5	10 [10-10]	10 [10-10]	>0.9999	0.5	10 [10-10]	10 [10-10]	>0.9999	0.5	10 [10-10]	10 [10-10]	>0.9999	0.5
**IL-13**	180 [10-315]	1055 [421-1676]	<0.0001	0.8716	10 [10-40]	241 [51-656]	0.0007	0.7863	10 [10-55]	245 [37-611]	0.0001	0.8157	90 [10-325]	263 [101-512]	0.029	0.6922
**IL-15**	10 [10-10]	10 [10-10]	>0.9999	0.5	10 [10-10]	10 [10-10]	>0.9999	0.5	10 [10-10]	10 [10-10]	>0.9999	0.5	10 [10-10]	10 [10-10]	>0.9999	0.5

**IL-17A**	20 [10-118]	80 [36-135]	0.0655	0.6627	25 [10-47]	28 [10-55]	0.8522	0.5167	10 [10-43]	35 [10-65]	0.4336	0.5667	10 [10-10]	11 [10-41]	0.0218	0.6765
**IL-21**	10 [10-10]	10 [10-10]	0.5237	0.5345	10 [10-10]	10 [10-10]	>0.9999	0.5	10 [10-10]	10 [10-10]	>0.9999	0.5167	10 [10-10]	10 [10-10]	>0.9999	0.5
**IL-23**	25 [25-25]	25 [25-25]	>0.9999	0.5	25 [25-25]	25 [25-25]	0.5025	0.5255	25 [25-25]	25 [25-25]	>0.9999	0.5167	25 [25-25]	25 [25-25]	>0.9999	0.5167
**IP-10**	160 [160-380]	8232 [2290-26225]	0.0003	0.7951	160 [160-160]	160 [160-36508]	0.0122	0.6794	160 [160-160]	11725 [940-102775]	<0.0001	0.8382	160 [160-12700]	19600 [2615-107695]	0.0004	0.7941
**MCP-1**	140 [140-2350]	1850 [140-15021]	0.1586	0.6147	140 [140-140]	140 [140-13529]	0.0714	0.6382	140 [140-2350]	140 [140-15021]	0.183	0.6059	140 [140-1270]	1170 [140-14174]	0.0894	0.6343
**MIG**	100 [100-12884]	4755 [100-17593]	0.0848	0.6451	100 [100-100]	100 [100-8275]	0.035	0.6559	100 [100-100]	921 [100-10117]	0.0085	0.7078	100 [100-1419]	163 [100-12189]	0.244	0.5951
**MIP-1α**	200 [140-1095]	18313 [6885-30448]	<0.0001	0.9559	3380 [140-29725]	9415 [140-29860]	0.4392	0.5686	140 [140-273]	3555 [380-16975]	0.0002	0.8059	140 [140-510]	490 [140-1468]	0.0266	0.6853
**MIP1-β**	130 [10-798]	2828 [1189-4604]	<0.0001	0.85	1550 [10-5505]	1843 [256-5431]	0.4351	0.5696	10 [10-350]	781 [10-2949]	0.0226	0.6931	10 [10-123]	117 [10-380]	0.0188	0.7
**RANTES**	10 [10-253]	1668 [832-5045]	<0.0001	0.9029	480 [10-1025]	813 [21-2266]	0.2358	0.6049	10 [10-10]	825 [10-1403]	0.0005	0.7804	10 [10-191]	160 [10-606]	0.0256	0.6892
**sCD40L**	10	318	<0.0001	0.9461	10	55	0.001	0.7588	10	55	0.0018	0.7441	10	41	0.0606	0.6549

	[10-13]	[194-521]			[10-10]	[10-149]			[10-10]	[10-261]			[10-30]	[10-93]		
**TNF-α**	110<br>[10-192]	1923<br>[1630-5130]	<0.0001	0.9598	350<br>[10-1123]	1753<br>[552-2655]	0.0271	0.6941	10<br>[10-15]	1068<br>[233-1890]	<0.0001	0.9314	10<br>[10-81]	328<br>[115-660]	<0.0001	0.8314

Results of the measured concentrations are reported as medians and 25th – 75th percentiles in infected and non-infected children. The degrees of significance of the differences between the concentrations measured in the two groups of children are reported as *p* values. The diagnostic ability of each cytokine was assessed by receiver operator characteristics (ROC) curve analysis and the areas under the curves are reported in the table.
*Mann-Whitney test
