# Supplementary material for: Specific Host Signatures for the Detection of Tuberculosis Infection in Children in a Low TB Incidence Country
Source: Front Immunol. 2021 Mar 15;12:575519. doi: 10.3389/fimmu.2021.575519 (PMC8005539; doi:10.3389/fimmu.2021.575519)
Supplement: Supplementary file 4 [file Table_4.pdf]

Supplementary Table 4. PPD-, ESAT-6-, CFP-10-, and HBHA-induced cytokines (validation cohort).

Host-marker	PPD				ESAT-6				CFP-10				HBHA			
	Median [P25-P75]		p*	Area under ROC curve	Median [P25-P75]		p*	Area under ROC curve	Median [P25-P75]		p*	Area under ROC curve	Median [P25-P75]		p*	Area under ROC curve
	Non-infected	Infected			Non-infected	Infected			Non-infected	Infected			Non-infected	Infected		
**IFN-γ**	69 [10-168]	20000 [15796-20000]	<0.0001	0.9788	10 [10-10]	2408 [117-9657]	<0.0001	0.92	10 [10-10]	2419 [167-20000]	<0.0001	0.9679	10 [10-33]	1000 [341-2405]	<0.0001	0.9719
**IP-10**	10 [10-745]	6976 [2415-15704]	<0.0001	0.85	50 [10-3156]	9989 [4549-37804]	<0.0001	0.8313	10 [10-620]	12414 [5542-56420]	<0.0001	0.9269	10 [10-1183]	10542 [3887-59989]	<0.0001	0.8425
**MIG**									10 [10-1445]	13441 [4263-42462]	<0.0001	0.8795	10 [10-911]	15783 [7946-24065]	<0.0001	0.8438
**MIP-1α**	49 [26-85]	2075 [332-2222]	<0.0001	0.8225	10 [10-10]	72 [12-344]	<0.0001	0.8475	10 [10-10]	140 [10-233]	<0.0001	0.8128	10 [10-222]	54 [29-122]	<0.0001	0.84
**TNF-α**	352 [149-637]	1188 [361-3574]	0.0013	0.7506	38 [10-61]	281 [78-628]	<0.0001	0.8469	10 [10-33]	247 [87-687]	<0.0001	0.8776	35 [11-96]	187 [101-573]	<0.0001	0.8344

Results of the measured concentrations are reported as medians and 25th – 75th percentiles in infected and non-infected children. The degrees of significance of the differences between the concentrations measured in the two groups of children are reported as *p* values. The diagnostic ability

of each cytokine was assessed by receiver operator characteristics (ROC) curve analysis and the areas under the curves are reported in the table.
*Mann-Whitney test
