# Supplementary material for: Specific Host Signatures for the Detection of Tuberculosis Infection in Children in a Low TB Incidence Country
Source: Front Immunol. 2021 Mar 15;12:575519. doi: 10.3389/fimmu.2021.575519 (PMC8005539; doi:10.3389/fimmu.2021.575519)
Supplement: Supplementary file 5 [file Table_5.pdf]

Supplementary Table 5. PPD-, ESAT-6-, CFP-10-, and HBHA-induced cytokines (exploratory cohort).

Host-marker	PPD				ESAT-6				CFP-10				HBHA			
	Median [P25-P75]		p*	Area under ROC curve	Median [P25-P75]		p*	Area under ROC curve	Median [P25-P75]		p*	Area under ROC curve	Median [P25-P75]		p*	Area under ROC curve
	LTBI	aTB			LTBI	aTB			LTBI	aTB			LTBI	aTB		
**GM-CSF**	5615 [1935-6520]	3864 [3625-6474]	0.9253	0.5116	2018 [337-4179]	546 [131-2235]	0.1369	0.6644	1138 [94-2890]	1128 [146-2902]	0.8512	0.5231	273 [94-460]	268 [72-407]	0.9419	0.5093
**IFN-γ**	19950 [17986-19985]	19979 [19873-19989]	0.4436	0.5856	19710 [2772-19981]	1989 [143-16009	0.1158	0.6736	4093 [264-19943]	6238 [52-19963]	0.9583	0.5069	1303 [431-3341]	320 [68-2286]	0.2151	0.6389
**IL-2**	10 [10-16]	10 [10-10]	0.5985	0.5602	10 [10-10]	10 [10-10]	0.3635	0.5486	10 [10-69]	10 [10-10]	0.0242	0.6944	10 [10-26]	10 [10-21]	0.4236	0.5764
**IL-6**	13020 [7060-38365]	15285 [7161-33683]	0.8918	0.52	6260 [948-35790]	403 [140-17865]	0.2155	0.6471	785 [283-5739]	2460 [330-6215]	0.6488	0.553	193 [140-398]	140 [140-245]	0.1706	0.6389
**IL-10**	10 [10-10]	10 [10-10]	>0.9999	0.5	10 [10-10]	10 [10-10]	>0.9999	0.5	10 [10-10]	10 [10-10]	>0.9999	0.5	10 [10-10]	10 [10-10]	>0.9999	0.5
**IL-13**	1200 [540-1764]	574 [267-1571]	0.341	0.6065	533 [62-929]	95 [34-248]	0.0458	0.7176	380 [24-720]	188 [55-440]	0.4823	0.5787	263 [184-374]	237 [36-613]	0.7466	0.537
**IL-15**	10 [10-10]	10 [10-10]	>0.9999	0.5	10 [10-10]	10 [10-10]	>0.9999	0.5	10 [10-10]	10 [10-10]	>0.9999	0.5	10 [10-10]	10 [10-10]	>0.9999	0.5
**IL-17A**	93	40	0.0298	0.7361	33	10	0.188	0.6412	41	15	0.3188	0.6065	28	10	0.0259	0.7269

	[65-167]	[16-99]			[10-94]	[10-39]			[10-70]	[10-46]			[10-63]	[10-25]		
**IL-21**	10 [10-10]	10 [10-10]	0.4975	0.5588	10 [10-10]	10 [10-10]	>0.9999	0.5	10 [10-10]	10 [10-10]	>0.9999	0.5278	10 [10-10]	10 [10-10]	>0.9999	0.5
**IL-23**	25 [25-25]	25 [25-25]	>0.9999	0.5	25 [25-25]	25 [25-25]	0.5034	0.5556	25 [25-25]	25 [25-25]	>0.9999	0.5278	25 [25-25]	25 [25-25]	>0.9999	0.5278
**IP-10**	9317 [4203-36075]	7050 [870-21400]	0.4878	0.5787	160 [160-19155]	1375 [160-83306]	0.1706	0.6389	13985 [160-105250]	8225 [3838-49550]	0.9833	0.5046	28240 [5320-186250]	13000 [1605-49750]	0.3835	0.5972
**MCP-1**	2980 [140-16175]	140 [140-14521]	0.5019	0.5718	1630 [140-13855]	140 [140-13771]	0.4454	0.787	2980 [140-16175]	140 [140-13771]	0.3449	0.5972	2980 [140-13855]	140 [140-14521]	0.7378	0.537
**MIG**	3530 [100-18978]	7260 [100-19417]	0.9079	0.5139	100 [100-11891]	100 [100-6538]	0.8704	0.5185	198 [100-7450]	6480 [100-12057]	0.2641	0.5278	4434 [100-13719]	100 [100-8434]	0.1698	0.6435
**MIP-1α**	23063 [7226-40360]	16313 [5410-28543]	0.4457	0.5856	27713 [1646-37860]	630 [140-17141]	0.0298	0.7361	3055 [380-19474]	3580 [270-15665]	0.9833	0.5046	565 [174-1468]	220 [140-2623]	0.4233	0.588
**MIP1-β**	2988 [1379-4604]	2278 [1036-5662]	0.6921	0.5463	2415 [1290-5915]	392 [14-2042]	0.0306	0.7361	428 [10-3178]	976 [26-2555]	0.8557	0.5208	137 [14-408]	85 [10-321]	0.6658	0.5486
**RANTES**	1605 [742-4884]	1845 [920-5245]	0.8512	0.5231	1133 [415-4013]	245 [10-1133]	0.0267	0.7407	568 [10-1425]	830 [33-1488]	0.6322	0.5532	155 [10-829]	175 [10-525]	0.8394	0.5231
**sCD40L**	325 [216-505]	303 [106-584]	0.8275	0.5255	72 [10-273]	10 [10-85]	0.0744	0.6898	23 [10-267]	108 [10-275]	0.5346	0.5671	41 [10-86]	28 [10-164]	0.8531	0.5208

**TNF-α**	1885 [1239-5368]	4104 [1763-5213]	0.253	0.6273	1753 [849-1943]	1751 [51-3748]	0.9833	0.5046	1068 [98-1609]	1119 [339-3323]	0.6534	0.5509	380 [10-680]	229 [120-784]	0.794	0.5301

Results of the measured concentrations are reported as medians and 25th – 75th percentiles in children with a latent TB infection (LTBI) and those with active tuberculosis (aTB). The degrees of significance of the differences between the concentrations measured in the two groups of children are reported as *p* values. The diagnostic ability of each cytokine was assessed by receiver operator characteristics (ROC) curve analysis and the areas under the curves are reported in the table. *Mann-Whitney test
