# Supplementary material for: Specific Host Signatures for the Detection of Tuberculosis Infection in Children in a Low TB Incidence Country
Source: Front Immunol. 2021 Mar 15;12:575519. doi: 10.3389/fimmu.2021.575519 (PMC8005539; doi:10.3389/fimmu.2021.575519)
Supplement: Supplementary file 6 [file Table_6.pdf]

Supplementary Table 6. PPD-, ESAT-6-, CFP-10-, and HBHA-induced cytokines (validation cohort).

Host-marker	PPD				ESAT-6				CFP-10				HBHA			
	Median [P25-P75]		p*	Area under ROC curve	Median [P25-P75]		p*	Area under ROC curve	Median [P25-P75]		p*	Area under ROC curve	Median [P25-P75]		p*	Area under ROC curve
	LTBI	aTB			LTBI	aTB			LTBI	aTB			LTBI	aTB		
**IFN-γ**	20000 [15893-20000]	20000 [9912-20000]	0.6557	0.535	2560 [84-16773]	1207 [117-9657]	0.7370	0.5325	1441 [67-19945]	7270 [266-20000]	0.3284	0.5921	1322 [662-6548]	483 [167-2288]	0.0524	0.68
**IP-10**	6910 [2415-13408]	8233 [2429-20123]	0.743	0.5313	7331 [4464-32908]	13340 [4787-37804]	0.3545	0.5875	12062 [3650-27598]	12414 [7708-73674]	0.4234	0.5763	9572 [3887-44010]	12566 [3748-68384]	0.6345	0.545
**MIG**									12750 [3035-41999]	14627 [5949-45629]	0.3838	0.5829	14030 [5599-45978]	15862 [10778-20668]	0.862	0.5175
**MIP-1α**	2181 [1120-2222]	1086 [196-2222]	0.1885	0.6188	158 [10-545]	49 [31-192]	0.93	0.5088	33 [10-449]	151 [10-233]	0.4518	0.5711	71 [36-241]	41 [12-98]	0.1457	0.635
**TNF-α**	1346 [865-3560]	463 [267-4495]	0.211	0.6175	333 [24-872]	258 [86-485]	0.7427	0.5313	250 [52-809]	247 [93-578]	0.9833	0.5026	333 [170-677]	120 [58-279]	0.0089	0.7388

Results of the measured concentrations are reported as medians and 25th – 75th percentiles in children with a latent TB infection (LTBI) and those with active tuberculosis (aTB). The degrees of significance of the differences between the concentrations measured in the two groups of children are reported as *p* values. The diagnostic ability of each cytokine was assessed by receiver operator characteristics (ROC) curve analysis and the areas under the curves are reported in the table. *Mann-Whitney test
