# Supplementary material for: Specific Host Signatures for the Detection of Tuberculosis Infection in Children in a Low TB Incidence Country
Source: Front Immunol. 2021 Mar 15;12:575519. doi: 10.3389/fimmu.2021.575519 (PMC8005539; doi:10.3389/fimmu.2021.575519)
Supplement: Supplementary file 7 [file Image_1.pdf]

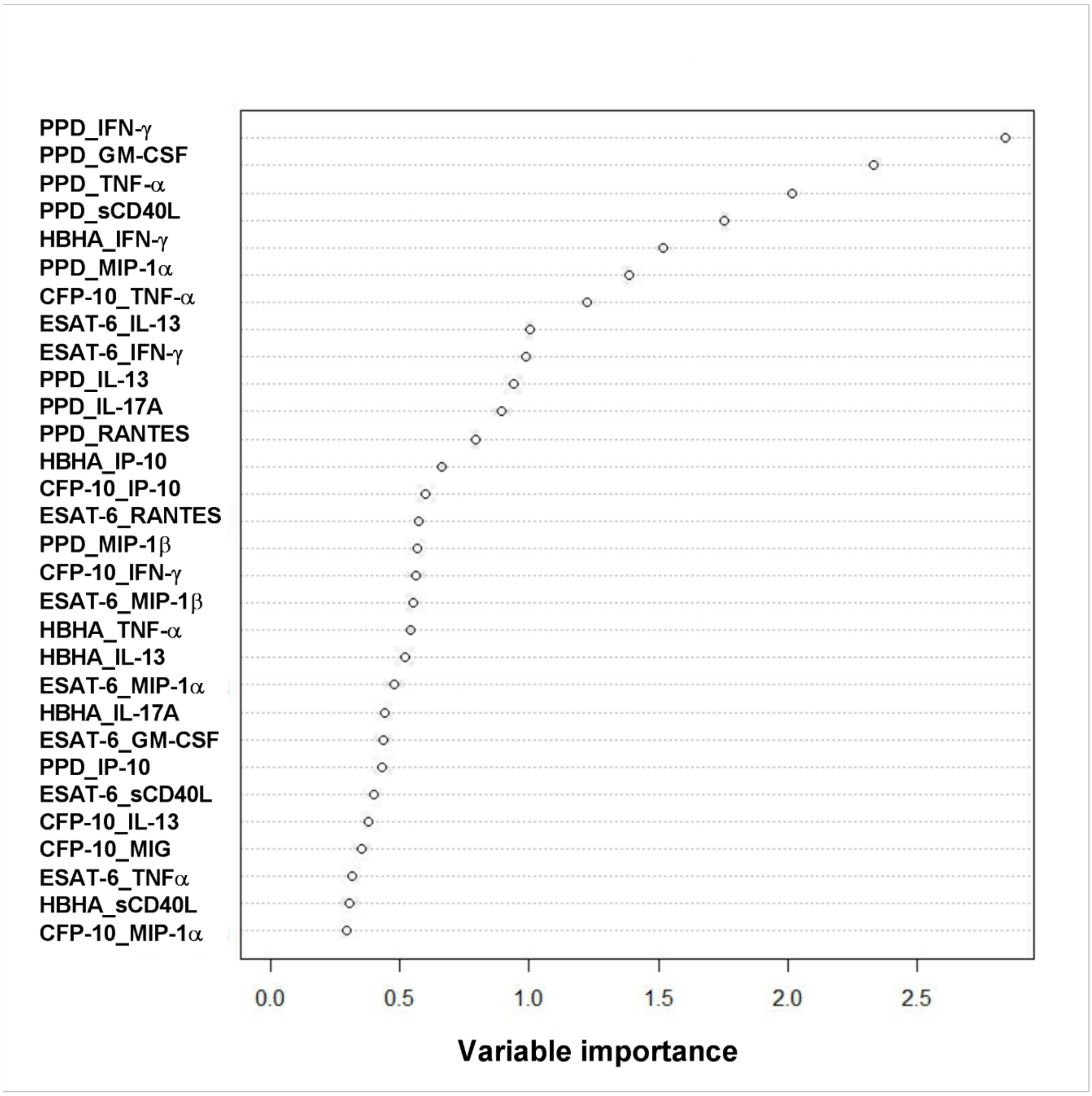

Supplementary Figure 1. Random forest analyses performed on PPD-, ESAT-6-, CFP-10-, and HBHA- host markers for *M. tuberculosis*-infected and non-infected children from the exploratory cohort.
