# Supplementary material for: Specific Host Signatures for the Detection of Tuberculosis Infection in Children in a Low TB Incidence Country
Source: Front Immunol. 2021 Mar 15;12:575519. doi: 10.3389/fimmu.2021.575519 (PMC8005539; doi:10.3389/fimmu.2021.575519)
Supplement: Supplementary file 8 [file Image_2.pdf]

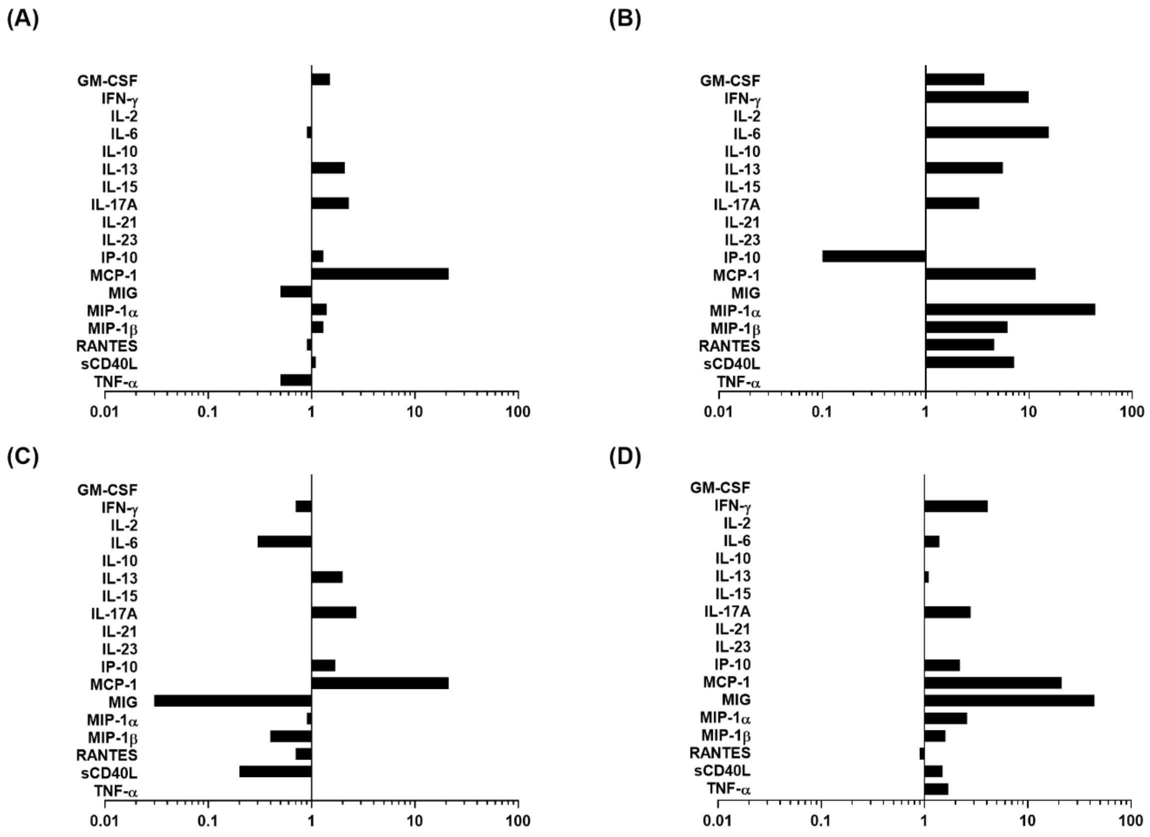

Supplementary Figure 2. Ratios between the concentrations (median value) of each host marker obtained for children with LTBI and children with aTB from the exploratory cohort. Results obtained in response to PPD, ESAT-6, CFP-10, and HBHA are represented on panels A, B, C and D, respectively. The host marker identification is indicated on the left side of each graph. Each ratio is represented by a horizontal bar. Ratios higher than 1, represented on the right parts of the graphs, indicate higher concentrations in children with LTBI compared to those with aTB, whereas ratios lower than 1, represented on the left sides of the graphs, indicate higher concentrations in children with aTB compared to those with LTBI.
